# Netherton Syndrome in Children: Management and Future Perspectives

**DOI:** 10.3389/fped.2021.645259

**Published:** 2021-05-10

**Authors:** Federica Barbati, Mattia Giovannini, Teresa Oranges, Lorenzo Lodi, Simona Barni, Elio Novembre, Ermanno Baldo, Mario Cristofolini, Stefano Stagi, Silvia Ricci, Francesca Mori, Cesare Filippeschi, Chiara Azzari, Giuseppe Indolfi

**Affiliations:** ^1^Immunology Unit, Department of Pediatrics, Meyer Children's University Hospital, Florence, Italy; ^2^Allergy Unit, Department of Pediatrics, Meyer Children's University Hospital, Florence, Italy; ^3^Dermatology Unit, Department of Pediatrics, Meyer Children's University Hospital, Florence, Italy; ^4^“Giovan Battista Mattei” Research Institute, Stenico, Italy; ^5^Endocrinology Unit, Department of Pediatrics, Meyer Children's University Hospital, Florence, Italy; ^6^Pediatric and Liver Unit, Meyer Children's University Hospital, Florence, Italy

**Keywords:** Netherton syndrome, management, immunology, dermatology, allergology, pediatrics

## Abstract

Netherton syndrome (NS) is a genetic, multisystemic disease classically distinguished by a triad of clinical manifestations: congenital ichthyosiform erythroderma, hair shaft abnormalities, and immune dysregulation. Due to the complex pathogenesis of the disease, there are no specific therapies currently accessible for patients with NS. An early diagnosis is crucial to start the correct management of these patients. A multidisciplinary approach, including specialists in immunology, allergology, and dermatology, is necessary to set up the best therapeutic pathway. We conducted a review with the aim to summarize the different therapeutic strategies currently accessible and potentially available in the future for children with NS. However, given the limited data in the literature, the best-tailored management should be decided upon the basis of the specific clinical characteristics of the patients with this rare clinical condition. Further comprehension of the pathophysiology of the disease could lead to more efficacious specific therapeutic options, which could allow a change in the natural history of NS.

## Introduction

Netherton syndrome (NS) is a rare, multisystemic, autosomal recessive disease described first by Comel in 1949 and later by Netherton in 1958 (1, 2). It is classically distinguished by a triad of clinical manifestations: congenital ichthyosiform erythroderma, hair shaft abnormalities, and immune dysregulation (3). The incidence of the disorder is evaluated to be 1/200,000, and the prevalence 1–9/1,000,000 (4). The prognosis of NS may be severe, with significant mortality in the first years of life due to potentially fatal complications. Skin and hair defects persist throughout life, but the disorder usually ameliorates with age (4).

NS results from mutations in the serine protease inhibitor Kazal-type 5 (SPINK5) gene encoding lymphoepithelial Kazal-type-related inhibitor (LEKTI), expressed in hair follicles and the granular layer of the epidermis (5). Deficiencies or abnormalities in LEKTI lead to the premature stratum corneum detachment and defect of skin barrier function, resulting from an excessive serine protease activity.

In the LEKTI-deficient epidermis of NS patients, the unrestricted activity of epidermal proteases triggers the secretion of proinflammatory interleukin (IL)-8, tumor necrosis factor-α (TNF-α), and proallergic cytokines (thymic stromal lymphopoietin, TSLP), through proteinase-activated receptor 2 (PAR2) signaling in keratinocytes (6). Furthermore, TNF-α and IL-1 increase the secretion of TSLP, which can act in synergy with these proinflammatory molecules to amplify pro-T helper-2 (Th2) cytokine expression by activated mast cells (7).

The phenotypic manifestations of NS vary widely. Bamboo hair (trichorrhexis invaginata), ichthyosis linearis circumflexa (ILC), erythroderma, and atopic predisposition with high serum levels of immunoglobulin E (IgE) are the typical clinical presentations of the disease (8, 9). Immunological alterations, such as B-cell immunodeficiency and selective antibody deficiency, have also been described in NS (10).

Life-threatening complications, including severe cutaneous infections up to sepsis and hypernatremic dehydration, are frequent in infancy. Dermopathic enteropathy with prolonged diarrhea and malnutrition, resulting in failure to thrive, has also been reported in patients with NS (11, 12).

Although the standard clinical triad should address a clear diagnosis of NS, it may be misdiagnosed because the triad is not always complete, and other disorders have similar findings. Some examples of the latter concept include non-bullous congenital ichthyosiform erythroderma, erythrodermic psoriasis, lamellar ichthyosis, primary immunodeficiencies (such as Hyper IgE syndrome or Wiskott–Aldrich syndrome), atopic or seborrheic dermatitis, and acrodermatitis enteropathy (4). Also, due to the complex pathogenesis of the disease, there are no specific therapies currently available for patients with NS (8).

This review aims to give a hint of the complexity of the management of the patients affected by NS and to briefly summarize the different therapeutic strategies accessible at the moment and potentially available in the future (Table 1).

**Table 1:** Summary of the different therapeutic strategies accessible and potentially available in the future for Netherton syndrome.

**Therapy**	**Reference(s)**
**Antiseptics**	(13–15)
Chlorhexidine, potassium permanganate, polyhexanide, octenidine, and diluted bleach baths	
**Emollients and keratolytics**	(13, 16–19)
**Topical corticosteroids**	(20, 21)
**Topical calcipotriol**	(22–25)
**Topical calcineurin inhibitors**	(26–32)
Pimecrolimus, tacrolimus	
**Topical protease inhibitors**	(33)
Kallikrein inhibitors	(34)
Recombinant alpha1-antitrypsin	(35, 36)
**Retinoids**	(37–40)
Acitretin, isotretinoin	
**Phototherapy**	(41–45)
Ultraviolet A1, psoralen plus ultraviolet, and narrowband ultraviolet B	
**Intravenous immunoglobulins**	(10, 17, 46–48)
**Future perspectives**	
Biologic drugs	(45, 49–54)
Infliximab, dupilumab, omalizumab, and ustekinumab	
Phage therapy	(55–58)
Gene therapy	(59–63)
**Management of complications and comorbidities**	(46, 64–86)

## Methods

**Figure 1:**
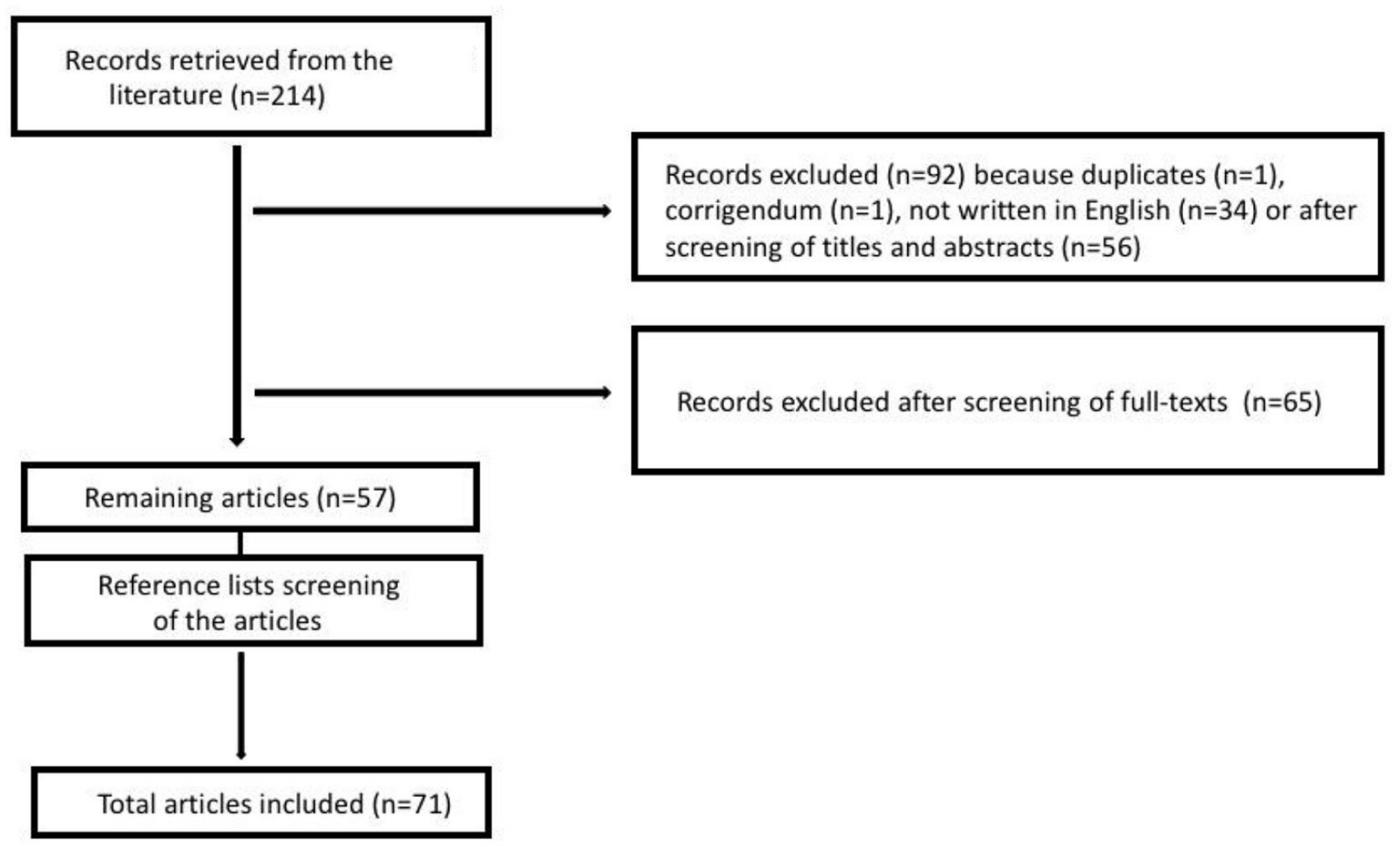
Flowchart of the research process.

We searched the PubMed database using the keywords “children” OR “child” OR “pediatr^*^,” associated with the keywords “Netherton” AND “syndrome,” up to April 25th, 2020. In order to access proper, international, and specific literature on the topic, articles were limited to English language, and they were excluded if they were redundant or not pertinent to the specific topic investigated based on the paper's title, abstract, or full text (Figure 1). Reference lists of all relevant articles were evaluated as well, looking for other potential pertinent articles. The process was independently carried out by two researchers (FB and MG).

## Results

### Antiseptics

In patients with NS, the use of antiseptics (2–3 times a week) may be useful to reduce the risk of recurrent skin infections, as suggested for congenital ichthyoses (13). Chlorhexidine (dilution 5/1,000–5/10,000), potassium permanganate (dilution 1/10,000), polihexanide 0.1%, octenidine 0.1%, or diluted bleach baths (0.005% solution) may be used and then rinsed to avoid skin irritation. Iodine-based products are not recommended (13–15).

### Emollients and Keratolytics

Emollients have been demonstrated to be useful in patients with skin barrier deficits as they act through skin hydration, lubrification, and occlusion. They should be applied several times a day, ideally after bathing (13).

Keratolytics remove scaling and hyperkeratosis, inducing an increase in stratum corneum turnover (16). In particular, 12% ammonium lactate lotion applied twice daily has been described by different authors to result in excellent amelioration of the skin clinical manifestations in many patients with NS, in the absence of long-term adverse effects (17–19). As suggested for congenital ichthyoses, all keratolytics should be avoided in young children and newborns; salicylic acid is contraindicated in children under the age of 2, and urea (≥10%) is not recommended in children under 1 year of age, except on limited areas once daily (13).

### Topical Corticosteroids

Topical corticosteroids are the cornerstone of therapy for various dermatoses due to their anti-inflammatory and antiproliferative properties (20). Patients with NS have impaired skin barrier function with augmented absorption of topical drugs. The transcutaneous absorption of corticosteroids (of both low- and high-potency) can rarely cause systemic side effects as growth retardation, hypertension, adrenal insufficiency, Cushing syndrome, or lethargy. Steroids should be monitored in patients with NS, and a low-dose steroid should only be used for a limited time in limited body areas. Specific experiences have been described in NS with hydrocortisone ointment treatment (21).

### Topical Calcipotriol

Calcipotriol is a synthetic vitamin D analog with an antiproliferative effect that promotes terminal differentiation of the epidermis in several skin disorders (22). Since keratinocyte proliferation is promoted and differentiation is inhibited in NS, calcipotriol could be useful as a topical treatment (23). For example, the maximum recommended dose for pediatric patients with psoriasis is 50 g/week between 6 and 12 years and 75 g/week over 12 years (24).

The application of 0.05% calcipotriol ointment every 4 days (<20 g/week/m^2^) in a pediatric patient with ILC led to the improvement of the erythema and scaling with nearly total remission after 3 weeks. Few lesions reappeared after 3–4 weeks, which responded well again to topical calcipotriol application. The treatment was well-tolerated without adverse effects such as hypercalcemia and hypercalciuria (25). The application of calcipotriol ointment (50 mcg/g) twice every day for 12 weeks in a girl with NS improved the skin roughness, but the degree of erythema worsened (22). The different clinical response to topical calcipotriol could be explained by differences in the proliferation of the epidermal cells, and it should be investigated on a more extensive group of patients with NS (25).

### Topical Calcineurin Inhibitors

Pimecrolimus and tacrolimus are calcineurin inhibitors: they bind calcineurin, inhibiting the production of proinflammatory mediators, including histamine, tryptase, eicosanoid, and cytokines, such as IL-4, IL-2, TNF-α, and interferon-γ. Pimecrolimus and tacrolimus have established effectiveness in the topical therapy of inflammatory skin diseases, e.g., atopic dermatitis (26). Tacrolimus has more pleiotropic targets, while pimecrolimus has a particular action on mast cells and T cells (27). Since patients with NS may be susceptible to increased transcutaneous absorption due to the epidermal barrier defect, monitoring the drug level in the blood, the renal and liver function closely, especially when using tacrolimus, is recommended (28, 29). Indeed, pimecrolimus cream has immunomodulating effects that are similar to those from tacrolimus but shows a dissimilar lipophilicity allocation, with a lesser epidermal permeation, as reported in the case of a boy with NS (30). Patients with NS who were treated with pimecrolimus responded rapidly, with improvements in itching and cutaneous manifestations and a reduction in disease severity. No clinical evidence of immune suppression was noted (31). The risk of cutaneous B-cell lymphoproliferative disease has been studied in the literature but it has been excluded among a large pediatric population with atopic dermatitis (32). In conclusion, based on the reported literature, the utilization of topical pimecrolimus and tacrolimus together with blood exam monitoring in patients with NS seems to be effective and mostly well-tolerated.

### Topical Protease Inhibitors

Serine proteases are expressed in the skin and are selectively inhibited by LEKTI function. The defective inhibitory activity against stratum corneum serine proteases may suggest an innovative therapeutic option for patients with NS that involves topical protease inhibitors (33).

#### Kallikrein Inhibitors

Skin proteases are inhibited by basic pH and zinc. Thus, an ointment containing zinc oxide and sodium bicarbonate could be used in patients with NS. The ointment is made by mixing 3 ml NaHCO_3_ with 5 g of 40% zinc oxide in lanolin and cod liver oil. With the application of this treatment four times a day for a week all over the body of a newborn with NS, an improvement of clinical manifestations and serum inflammatory markers were demonstrated. After the treatment, a reduction in hypertension, hypernatremia, and alkalosis was also reported, and this was considered a result of systemic kallikrein inhibition due to ointment use, as the patient did not receive any other systemic therapy (34).

#### Recombinant Alpha1-Antitrypsin

LEKTI could be implicated in skin desquamation due to its inhibitory action on trypsin and chymotrypsin enzymes of the stratum corneum. Alpha1-antitrypsin (AAT) is a serine protease inhibitor that could be employed as a replacement treatment for LEKTI. Indeed, it inhibits pancreatic trypsin, which is similar to stratum corneum trypsin and chymotrypsin enzymes (35). Even if a randomized, double-blind study found no statistically significant difference between placebo and 2% topical recombinant AAT (rAAT) gel applied twice daily for 21 days in pediatric patients with NS, rAAT could be investigated further in the future as a therapy for NS (36).

### Retinoids

Oral retinoids have been used to treat patients with NS with different success. Leung et al. (37) reported that a low dose (0.25 mg/kg for 6 months followed by 0.12 mg/kg for other 6 months) of oral acitretin was efficacious in the management of the skin signs and symptoms in a boy with NS, confirming the gradual remission of the skin lesions described by other authors with both acitretin and isotretinoin (38, 39). However, other published data showed that oral isotretinoin worsened cutaneous clinical manifestations in a girl affected by NS (40). Therefore, the role of oral retinoids in the treatment of NS is currently not clear, and further studies will be needed to elucidate its potential role in this disease.

### Phototherapy

Phototherapy [ultraviolet A 1 (UVA1), psoralen plus ultraviolet A (PUVA) and narrowband ultraviolet B (NB-UVB)] can be used in the long-term management of psoriasis and various dermatological conditions such as ichthyosis (41). The specific mechanism of function of phototherapy in NS is not known. However, it has been proposed that phototherapy may increase the production of serine protease inhibitors that balance the function of the defective LEKTI (42). Additionally, it may have immunomodulatory and apoptosis-inducing effects on keratinocytes, dendritic cells, mast cells, and T-lymphocytes (43). Dose-dependent and long-term side effects of phototherapy are an augmented risk of accelerated skin aging, lentigo formation, actinic keratosis, unusual cutaneous pigmentation, and ophthalmologic disorders. PUVA might raise the risk of skin cancers (melanoma and non-melanoma) (44). In a case report of a newborn with NS, treated with a total of 170 sessions of NB-UVB phototherapy, with an aggregate dose of 240 J over 3 years, there was the appearance of nevi and multiple pigmented lesions (41). In another case, of a boy treated with NB-UVB three times a week for a total of 30 sessions and an aggregate dose of 54.7 J/cm^2^, no side effects were reported, and the treatment led to significant improvement of the lesions (42).

Short-term NB-UVB therapy may be used in selected cases, but in NS patients, PUVA therapy and long-term UVB therapy are not recommended due to the increased risk of skin cancers (45).

### Intravenous Immunoglobulins

NS may respond to treatment with intravenous immunoglobulins (IVIG) as it has been described as a primary immunodeficiency disorder (46). Indeed, hypergammaglobulinemia and hypogammaglobulinemia have been reported in children with NS, but most of them have normal immunoglobulin levels (17). IVIG are used as replacement therapy in immunodeficiency disorders with antibody deficiency, but recently also with anti-inflammatory and immunomodulatory applications (47). In patients with selective antibody deficiency, prophylactic antibiotics and IVIG therapy can be effective in preventing infections (10). Small et al. (48) reported a decrease of the erythema, pustulation, scale, and pruritus in two pediatric patients that received IVIG 500 mg/kg monthly, already after three administrations. In children with NS, an immunologic evaluation should be considered and a trial of IVIG may be a reasonable therapeutic option in case of serious disease (48).

### Future Perspectives

While in children, the possible treatments are very limited, more trials have been conducted to find out new therapeutic strategies for NS in adults.

#### Biologic Drugs

Infliximab, a chimeric monoclonal antibody that specifically binds TNF-α, has been reported to be beneficial in two young women with NS, demonstrating the joint inflammatory pathogenesis between psoriasis and NS (49, 50). Infliximab could reduce skin inflammation in NS, decreasing the expression of TSLP, IL-6, and IL-8, but the treatment is not recommended considering the risk of skin cancers and recurrent infections reported in patients (45).

A few studies reported dupilumab as a possible treatment for NS that can improve signs and symptoms such as pruritus and scaling both in adults and children (51, 52). Dupilumab is an entirely human monoclonal antibody against the IL-4α receptor subunit. Thus, it blocks the signaling from IL-4 and IL-13, which are essential cytokines in the Th2 pathways and of paramount importance in atopic diseases, including atopic dermatitis and asthma (52).

Omalizumab, a humanized monoclonal antibody that specifically binds IgE, was reported to decrease allergic skin clinical manifestations in a 20-year-old male with NS (53).

Finally, a recent case report demonstrated clinical effect with substantially improving skin signs and symptoms in a 15-year-old girl with NS who received ustekinumab treatment, an entirely human monoclonal antibody against IL-12 and IL-23, which use is set up in the therapy of several clinical conditions including psoriasis. While IL-17 was found to be a dominant immune profile in NS, the authors took into consideration influencing the IL-17/IL-23 axis as an effective interventional approach. Therefore, therapy with an IL-17 inhibitor might be another possible strategy of treatment for patients with NS (54).

#### Phage Therapy

Bacteriophage therapy is an alternative therapy used to treat bacterial infections using bacterial viruses highly specific to their hosts. It could be an effective treatment to control chronic infections in patients with genetic diseases, such as cystic fibrosis (55) or NS who have a predisposition toward infections and can develop bacterial resistance and drug allergies. Phage can be applied in cream and liquid topic vehicles or as an oral therapy (56). A possible issue could be the potential modifications in bacterial phage sensitivity. If resistance is noted, it can usually be faced by using another preparation or an individual phage preparation. The response of the immune system to phage after extensive utilization could be a possible challenge. However, recent data from the literature reported that this might not represent an essential issue with a therapeutic regimen (57). The benefits of phage therapy are the capacity to deal with many antibiotic-resistant pathogens or antibacterial therapy in cases of drug allergy. Furthermore, it demonstrated little or no side effects with high tolerability (58). Therefore, phage therapy may represent an alternative to control infectious complications of a rare genetic disease, reducing the severity of NS and improving the patient's quality of life.

#### Gene Therapy

Gene-based therapies are being developed for the most debilitating and severe diseases as the molecular basis of inherited skin disorders becomes known (59). Recent studies demonstrated that gene therapy could be an effective treatment of genodermatosis (60). Therefore, NS is a potential disorder for gene treatment since its clinical manifestations appear to depend on the residual function of LEKTI. Thus, a partial augment in LEKTI activity might cause an improvement in the clinical manifestations. Roedl et al. built a recombinant viral vector-based on adeno-associated virus type 2, which expressed the functional cDNA of human SPINK5. Then, human keratinocytes from four unrelated pediatric patients with NS were transduced. A five-fold increase in mRNA expression of SPINK5 *in vitro* was demonstrated from the gene transfer. These findings showed how the gene transfer of SPINK5 into NS-keratinocytes with LEKTI-deficiency could be a future approach to an appropriate treatment of NS (61). Keratinocytes, including their stem cells or their derivatives, could be *in vitro*-cultured and epithelium sheets could be created for grafting. No published evidence reported immunological reaction to the graft or adverse events mediated by the vector in the *ex-vivo* humanized mouse skin engraftment model. Therefore, the replication-defective viral vectors could efficaciously address stable gene transfer to keratinocyte stem cells in the absence of evidence of toxicity (62). Di et al. generated three gene-reshaped epithelial sheets for autografting, highlighting the usefulness of lentiviral gene variation of primary keratinocytes. In a 27-year-patient with NS, the lentiviral delivery processes utilized for transducing primary keratinocytes and grafting of a reshaped autologous epithelial sheet were reported to be secure and useful. Nonetheless, identification, isolation, and variation of essential keratinocyte stem cell populations are needed to guarantee their long-lived engraftment and constant protein expression (63).

### Management of Complications and Comorbidities

In NS, meticulous surveillance of the fluid balance is essential because the imperceptible fluid loss through the skin and the enteric tract may lead to decompensation of the delicate fluid stability. Dehydration can lead to severe hypernatremia that needs to be treated promptly because it can result in a fatal outcome (64). Neurologic signs and symptoms have been ascribed to the toxic effects of hypernatremia in a few cases of NS (65). Pohl et al. (66) reported a case of acute bilateral renal vein thrombosis as a complication of hemoconcentration.

A few cases of neonatal respiratory insufficiency and pulmonary hypertension have been described in NS. Macknet et al. (67) reported an infant who received extracorporeal membrane oxygenation treatment for persistent pulmonary hypertension that was probably secondary to bronchopneumonia due to the thickness of the amniotic fluid enclosing exfoliated epidermal cells. Okulu et al. (68) described a newborn who required invasive respiratory support, surfactant and bronchodilators due to respiratory insufficiency and mild pulmonary hypertension.

Renner et al. (46) reported the possibility of a deficient response to vaccination in pediatric patients with NS. Thus, the latter event should be taken into account when assessing their vaccination schedule.

Children with NS that have developed acute pancreatitis have been described. The treatment consisted of intravenous antibiotics and pancreatic enzymes, and the clinical manifestations rapidly improved. The association between pancreatitis and NS has been explained with different hypotheses. Both pancreatitis and NS have been linked to the LEKTI alterations, which lead to altered inhibition of serine proteases in both pancreatic and epidermal tissues (69). Another hypothesis is that an elevated serum level of IgE could possibly cause an immune response in the pancreas, triggering pancreatitis (70).

NS can be associated with food allergies, which may be severe (71, 72) and need proper management (73–76). Specific oral tolerance induction could be used with an improved outcome in these patients in which an allergy is included in a broader genetic disorder (77, 78).

In patients with digestive clinical manifestations, endoscopies should be considered because eosinophilic esophagitis might be a trait of NS and could improve with a food elimination diet (79).

Growth retardation and growth abnormality up to dwarfism have been reported in patients with NS (80, 81). Growth hormone (GH) deficiency has been described in a few cases (82). As an underlying mechanism, a lack of inhibition of proteases in the pituitary gland has been proposed, leading to the over-processing of GH. Aydin et al. (83) reported three pediatric cases with GH deficiency with subsequent good response to exogenous GH therapy.

In pediatric patients suffering from NS, barrier skin defect and immune dysregulation lead to recurrent cutaneous infections, including severe ones up to sepsis. Thus, prompt treatment, including the appropriate antimicrobial agent, is of critical importance to control these events (84).

Pediatric patients with NS also have an augmented risk for vitamin D deficiency, which should be supplemented (85). Finally, NS patients can show psychosocial and neuropsychological problems. Thus, in order to recognize these issues at an early phase, a standard follow-up is needed (86).

## Conclusion

NS is a rare genetic multisystemic disease for which an effective etiological treatment is not yet available. An early diagnosis is crucial to start the correct management for these patients. Genetic counseling and molecular prenatal diagnosis are feasible in families with a history of NS (87).

Usually, trichorrhexis invaginata, which is a pathognomonic feature of NS, suggests the diagnosis; otherwise, it could be delayed until its appearance. Thus, hair inspection should be performed at an early stage in order not to miss the diagnosis. Then, given the limited data in the literature, the best-tailored management should be decided upon the basis of the specific clinical characteristics of the patients with this rare clinical condition. A multidisciplinary approach, including specialists in immunology, allergology, and dermatology, is necessary to set up the best management and therapeutic pathway. Further comprehension of the pathophysiology of the disease could lead to more effective specific therapeutic options in the future, which could allow a change in the natural history of NS.

## Author Contributions

MG and EB conceptualized the work. MG and FB were responsible for literature search. FB, MG, TO, LL, and SB drafted the manuscript. FB, MG, TO, LL, SB, EN, EB, MC, SS, SR, FM, CF, CA, and GI analyzed, interpreted the data and critically revised the manuscript. All authors approved the final version of the manuscript as submitted and agreed to be accountable for all aspects of the work.

## Conflict of Interest

CF received an honorarium from Sanofi-Regeneron for congress talks. The remaining authors declare that the research was conducted in the absence of any commercial or financial relationships that could be construed as a potential conflict of interest.
